# C/EBP homologous protein (CHOP) mediates neuronal apoptosis in rats with spinal cord injury

**DOI:** 10.3892/etm.2012.745

**Published:** 2012-10-12

**Authors:** ZHANGFU WANG, CHUANYI ZHANG, ZHENGHUA HONG, HAIXIAO CHEN, WEIFU CHEN, GUOFU CHEN

**Affiliations:** Department of Orthopedics, Taizhou Hospital of Zhejiang Province, Taizhou, Zhejiang 317000, P.R. China

**Keywords:** C/EBP homologous protein, neuron, spinal cord injury, apoptosis

## Abstract

Spinal cord injury (SCI) is a severe health problem and the mechanism involved remains elusive. The aim of the present study was to elucidate the role of C/EBP homologous protein (CHOP), a prominent protein of the endoplasmic reticulum (ER) stress-mediated apoptosis in SCI. A total of 20 adult male Sprague-Dawley rats were divided into two groups at random, ten rats were subjected to a modified Allen’s test (using a weight-drop device) to induce a SCI model and the remaining ten rats only had the corresponding vertebral lamina removed with no injury and served as the sham-operated group. Pathological changes in the spinal cord were observed 12 h after injury by hematoxylin and eosin staining and TUNEL staining was performed to visualize apoptotic cells. The expression of CHOP was also detected by immunohistochemistry and quantitative real-time reverse transcription-polymerase chain reaction. The results showed that a typical apoptotic morphology, namely the increased the number of TUNEL-positive cells in the injured spinal cord. The expression levels of CHOP in the rats with SCI were increased compared with the sham-operated rats (P<0.05). These results revealed that CHOP-mediated ER stress-induced apoptosis may be involved in SCI.

## Introduction

Spinal cord injury (SCI) is a severe health problem worldwide, and it often causes enormous physical and mental pain to the patient and family (1–3). Normally, the primary injury is the mechanical impact afflicted directly on the spine, while the secondary injury to the spinal cord involves a number of self-destructive processes that occur by a variety of factors based on disturbances in ionic homeostasis, local edema, focal hemorrhage, excitotoxicity, presence of free radicals and free fatty acids (4–6). Previous studies have indicated that the TUNEL-positive glial cells and neurons within the lesion area are present at all stages studied between 4 h and 14 days, with a maximum presence between 8 h to 24 h, and spread to at least a few millimeters around the center of the lesion (7). However, the exact mechanism of cell death has not been fully clarified in nervous system injury (8). Therefore, identifying the specific molecular pathway mediating the apoptosis after SCI would be of great value to the patients concerned.

Certain studies have indicated that endoplasmic reticulum (ER) dysfunction leads to the activation of unfolded protein response under any destructive stimulus (9,10). When stress is present, the expression of chaperones is activated, protein translation is attenuated and ER-associated degradation is activated (11,12). Eventually, the cell may undergo apoptosis under a prolonged ER stress environment (13). Growth arrest and DNA damage 153/C/EBP homologous protein (CHOP) is a prominent protein involved in the pathway of ER stress-mediated cell apoptosis (14,15). CHOP-mediated ER stress induces cell death and is involved in several neurodegenerative diseases (16,17). Therefore, we hypothesized that increasing the expression of CHOP can prolong injury after SCI.

## Materials and methods

### Animal model induction

Twenty Sprague-Dawley (SD) male rats (Experimental Animal Center of Zhejiang University Hangzhou, China) were divided into two groups at random, the sham-operated group and the SCI group. Ten rats were assigned to the SCI group and spinal cord contusion injuries were inflicted by modified Allen’s method (using a weight of 10 g dropped from a height of 50 mm on the exposed spinal cord to produce a moderate contusion) after the T10 spinous process and the corresponding vertebral lamina were removed. The remaining ten rats received only T10 laminectomies and were not injured, and served as the sham-operated group. Following SCI, the rats had their bladders expressed at least twice a day. A test of the locomotor activities of both groups was carried out using an open-field locomotor scale, described by Basso, Beattie and Bresnahan (BBB) from complete paralysis (score 0) to normal locomotion (score 21) 12 h after SCI (18). All procedures were carried out according to the National Institutes of Health Guide for the Care and Use of Laboratory Animals (NIH Publications). Furthermore, all efforts were made to minimize the number of animals used and their suffering.

### Histological staining, TUNEL staining and immunohistochemistry

Five rats per group were anaesthetized 12 h after SCI by intraperitoneal injection of a lethal dose of Nembutal. The animals were perfused with 100 ml of normal saline and 250 ml of 4% formaldehyde by aortic cannulation for 20–30 min. The injured spinal cords of each rat were embedded in paraffin, and transverse paraffin sections (8-*μ*m thick) were mounted on silane-coated slides.

TUNEL staining was performed to visualize apoptotic cells in the injured spinal cord according to the manufacturer’s instructions. The cells labeled with trypan blue were counted under a microscope.

For immunohistochemistry, the sections were washed in 0.01 M PBS containing 0.3% Triton X-100 (pH 7.4, PBS-T), immersed in 2% normal horse serum in PBS for 120 min at 37°C, incubated overnight at 4°C with polyclone CHOP antibody (1:100, Santa Cruz Biotechnology, Santa Cruz, CA, USA) in PBS containing 1% bovine serum albumin and washed in PBS (3×5 min). The sections were incubated in biotinylated IgG (1:200; Boster Biotechnology, Wuhan, China) in PBS for 2 h at room temperature and washed in PBS-T (3×5 min). The sections were then incubated in avidin-biotin-peroxidase complex solution (1:100; Boster Biotechnology) for 2 h at room temperature and then rinsed in PBS-T (3×5 min). Visualization was achieved by incubating the tissue for 10 min in 0.04% 3-diaminobenzidine containing 0.01% H_2_O_2_. Rat immunoglobulin G (IgG) (1:200; Biomeda Corp., Foster City, CA, USA) was used instead of a primary antibody as the negative control.

### Quantitative real-time reverse transcription-polymerase chain reaction (qRT-PCR) analysis

The remaining five rats per group were re-anesthetized and decapitated 12 h after SCI. Total RNA of the lesion epicenter around the segment at T8 was obtained using the RNA extraction kit (Qiagen, Hilden, Germany) following the manufacturer’s instructions. For reverse transcription, RNA concentration was measured spectrophotometrically and 2 mg total RNA was added to the cDNA synthesis reaction system (20 ml). The reaction mixture consisted of 4 *μ*l 5X RT buffer, 2.5 *μ*mol/l oligo(dT), 5 mmol/l deoxyribo-nucleotide triphosphates and 20 U RNAasin (RNase inhibitor). The hexamers were annealed by incubating the samples at 70°C for 5 min. M-MLV reverse transcriptase of 200 U was added and then incubated at 42°C for 60 min and 72°C for 10 min. For qRT-PCR analysis, the reaction mixture (40 ml) consisted of 4 ml cDNA, 35.2 *μ*l SYBR-Green PCR mix, 0.5 *μ*l 5 U Taq DNA polymerase and 0.3 *μ*l 20 pmol/ml CHOP primer. The cDNA was denatured to 94°C for 3 min. The template was amplified for 40 cycles (denaturation at 94°C for 10 sec, annealing at 57°C for 30 sec and extension at 72°C for 30 sec), before collecting fluorescence at 72°C. Primers were used for the housekeeping gene, glyceraldehyde-3-phosphate dehydrogenase (GAPDH), in qRT-PCR to amplify GAPDH (forward, 5′-GGTGGACCTCATGGCCTACAT-3′; reverse, 5′-GCCTCT CTCTTGCTCTCAGTATCCT-3′), as an internal control, and CHOP (forward, 5′-CGGAGTGTACCCAGCACCATCA-3′; reverse, 5′-CCCTCTCCTTTGGTCTACCCTCA-3′).

### Statistical analysis

The sections were examined at ×400 magnification with UTHSCSA Image Tools 3.0 (University of Texas Medical School, San Antonio, TX, USA). The number of the positive cells was determined. Values are expressed as the means ± SD. The significant difference was calculated by a two-tailed Student’s t-test. P<0.05 was considered to indicate a statistically significant result. The expression of mRNA were calculated by fold change and estimated using the comparative CT method (2^−ΔΔCT^) normalizing to GAPDH CT values and relative to control samples.

## Results

### Successful induction of SCI animal model

**Figure 1:**
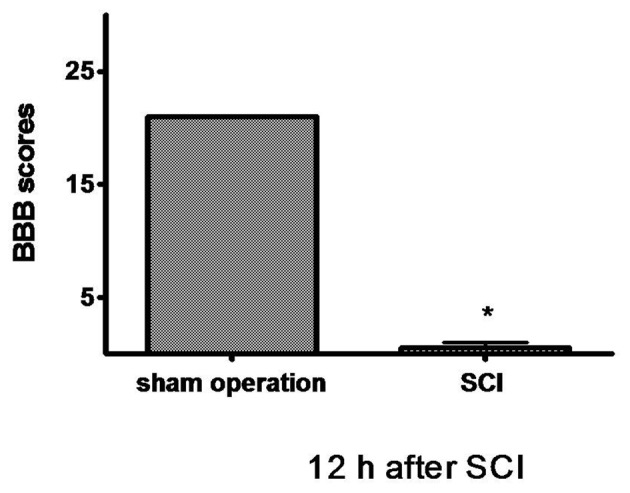
BBB scores in the two groups at 12 h after SCI. ^*^P<0.01, versus sham-operated group. SCI, spinal cord injury.

The animals in the SCI group demonstrated extreme and bilateral hind limb paralysis with no movement (score 0) or only slight movements of a joint (score 1) from the first hours post-injury when observed during open-field experimentation. The sham-operated group animals walked normally after recovery from the anesthesia. We scored animal locomotor activity according to the BBB scale 12 h after SCI, and the BBB score revealed that rats with SCI could no longer move (score 0–1) while the sham-operated rats walked normally (score 21). There were significant differences between both groups (P<0.01; Fig. 1).

### Pathological changes and TUNEL assay of injured spinal cord

**Figure 2:**
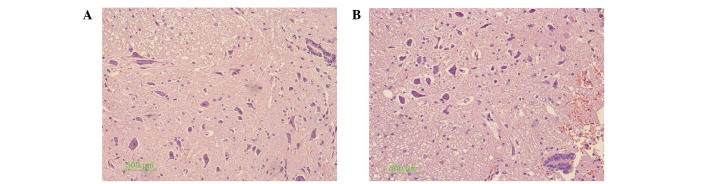
Pathological changes in the spinal cord in the two groups visualized by hematoxylin and eosin staining (×400 magnification). (A) The sham-operated rat group, (B) the rats with SCI group. SCI, spinal cord injury.

For histological evaluation, transverse sections of injured spinal cords were examined. Blood cells, dead neurons and an increased number of gliocytes were observed 12 h after injury in the injured spinal cord. The lesion center was characterized by the destruction of gray and white matter. A large cavity involving the dorsal and partially the lateral funiculus extended for at least 2 mm while the tissue was integrated. The neurons in the spinal cord of the sham-operated group had normal morphology, with a clear cytoplasm, and uniform and clear nuclei (Fig. 2).

**Figure 3:**
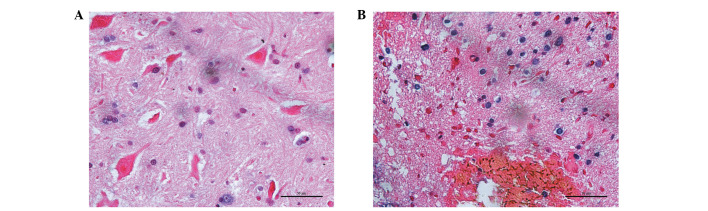
TUNEL-positive cells in the spinal cord in the two groups (×400 magnification). (A) The sham-operated rat group, (B) the rats with SCI group. SCI, spinal cord injury.

A few TUNEL-positive trypan blue cells were observed 12 h after injury in the sham-operated rats. Numerous TUNEL-positive trypan blue cells were present in the gray matter, confined to the lesion area surrounding the cavity. Morphological analysis indicated that a number of these TUNEL-positive cells were neurons and others were glial cells. These TUNEL-positive neurons typically exhibited a reduction of the cytoplasm and nucleus, creating pericellular space and nuclear fragmentation. Apoptotic changes in presumptive glial cells were also observed after injury. The majority of these TUNEL-positive glial cells were located within the lesion area, although several were present in the neighboring white matter. The cells typically exhibited small, fragmented nuclei with scarce visible cytoplasm surrounding them (Fig. 3). The percentage of TUNEL-positive cells within the lesion area in the rats with SCI was increased by 72.3±12.6% while compared with the normal rats 3.2±1.5% (P<0.01).

### Expression of CHOP in the spinal cord

**Figure 4:**
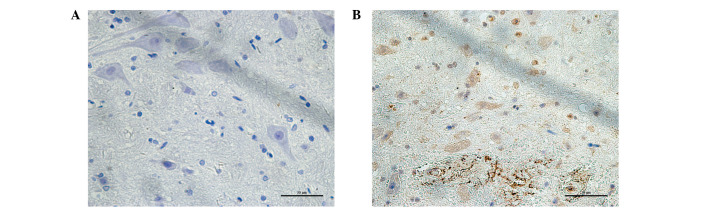
CHOP-positive cells in the spinal cord in the two groups (×400 magnification). (A) the sham-operated rat group, (B) the rats with SCI group. SCI, spinal cord injury.

The immunoreactivity of the CHOP protein was visualized as a granular immunostaining pattern. CHOP protein was expressed predominantly in the nucleus. Compared with the control group, CHOP-positive neurons were significantly increased in the injured spinal cord (Fig. 4). Quantitative analysis of the number and optical density of CHOP-positive neurons was increased compared with the sham-operated rats (P<0.01; Table I). The levels of CHOP mRNA in the injured spinal cord of rats with SCI were also increased 3.3±0.17 fold compared with the sham-operated group by qRT-PCR assay (P<0.01).

## Discussion

Previous studies have demonstrated that SCI is a process involving various self-destructive processes that occur by a variety of factors based on disturbances in ionic homeostasis, local edema, focal hemorrhage, excitotoxicity, presence of free radicals and free fatty acids (5,6). Certain studies have indicated that neuronal and glial cell apoptosis plays a role in SCI and that the inhibition of neuronal and oligodendroglial apoptosis may be a therapeutic strategy (19,20). The results from the present study demonstrated that the pathological changes of the neurons and TUNEL-positive cells increased in the injured spinal cord, which led to neuronal and glial cell loss, and finally induced the impairment of locomotor activity according to the BBB scale.

Two major pathways are involved in apoptosis induction: the extrinsic pathway which is activated by the plasma membrane death receptor ligation, and the intrinsic pathway which is activated by the mitochondria and other organelles, including the ER, Golgi apparatus and lysosomes (21–23). Experimental results provide evidence that the ER is the site of complex processes, including calcium storage, synthesis and folding of proteins and cell response to stress. ER function is impaired in numerous acute and chronic diseases of the brain, which in turn induce calcium store depletion and conserved stress responses (24). ER stress is present in physiological and pathological conditions, cellular injuries, tissue ischemia and amyotrophic lateral sclerosis (25–27). Certain studies, however, have suggested that the ER stress-signal may have a direct role in promoting cell death in neuronal injury diseases (26,28). CHOP plays a critical role in ER stress-induced apoptosis, and it is believed to play a central role in ER stress-induced cell death (29). CHOP has been implicated in mediating neurode-generation in animals with Alzheimer’s disease (30). CHOP activation has been observed in neurons undergoing apoptosis due to perturbations in ER calcium levels in an *in vivo* neurotoxin model of parkinsonism (17). Our results suggest a crucial role for CHOP in SCI-induced apoptosis of injured SCI. SCI may induce ER stress by the generation of free radicals, breakdown ionic homeostasis and excitotoxicity. If these effects were not counterbalanced, then the ER would be overwhelmed and initiate apoptosis as the ultimate ER stress. The present results provide a comprehensive view of the activation of ER stress pathways following SCI.

In conclusion, this study demonstrates that SCI may damage the ultrastructure of the spinal cord and cause locomotor activity impairment, and that CHOP plays a role in ER stress-mediated apoptosis in the injured spinal cord. These results may aid the design of potential therapeutic interventions for SCI.

## Figures and Tables

**Table I:** CHOP-positive cells in the spinal cord of the two groups.

	CHOP-positive neurons	
Group	Number (/mm^2^)	Optical density
Sham-operated group	1.3±0.7	132.5±18.8
SCI group	8.9±2.8^a^	186.2±21.6^a^

a P<0.01 compared with the sham-operated group. SCI, spinal cord injury; CHOP, C/EBP homologous protein.
